# Multidrug-Resistant Bacteria Isolated from Different Aquatic Environments in the North of Spain and South of France

**DOI:** 10.3390/microorganisms8091425

**Published:** 2020-09-16

**Authors:** Lara Pérez-Etayo, David González, José Leiva, Ana Isabel Vitas

**Affiliations:** 1 Department of Microbiology and Parasitology, University of Navarra, 31008 Pamplona, Spain; dgonzalez@unav.es (D.G.); avitas@unav.es (A.I.V.); 2 IdiSNA, Navarra Institute for Health Research, 31008 Pamplona, Spain; 3 Microbiology Service, Clínica Universidad de Navarra, University of Navarra, 31008 Pamplona, Spain; jleiva@unav.es

**Keywords:** WWTPs, collectors, rivers, antibiotic resistance bacteria, antibiotic resistance gene, ESBL

## Abstract

Due to the global progress of antimicrobial resistance, the World Health Organization (WHO) published the list of the antibiotic-resistant “priority pathogens” in order to promote research and development of new antibiotics to the families of bacteria that cause severe and often deadly infections. In the framework of the One Health approach, the surveillance of these pathogens in different environments should be implemented in order to analyze their spread and the potential risk of transmission of antibiotic resistances by food and water. Therefore, the objective of this work was to determine the presence of high and critical priority pathogens included in the aforementioned list in different aquatic environments in the POCTEFA area (North Spain–South France). In addition to these pathogens, detection of colistin-resistant Enterobacteriaceae was included due its relevance as being the antibiotic of choice to treat infections caused by multidrug resistant bacteria (MDR). From the total of 80 analyzed samples, 100% of the wastewater treatment plants (WWTPs) and collectors (from hospitals and slaughterhouses) and 96.4% of the rivers, carried antibiotic resistant bacteria (ARB) against the tested antibiotics. Fifty-five (17.7%) of the isolates were identified as target microorganisms (high and critical priority pathogens of WHO list) and 58.2% (*n* = 32) of them came from WWTPs and collectors. Phenotypic and genotypic characterization showed that 96.4% were MDR and resistance to penicillins/cephalosporins was the most widespread. The presence of *bla* genes, KPC-type carbapenemases, *mcr-1* and *vanB* genes has been confirmed. In summary, the presence of clinically relevant MDR bacteria in the studied aquatic environments demonstrates the need to improve surveillance and treatments of wastewaters from slaughterhouses, hospitals and WWTPs, in order to minimize the dispersion of resistance through the effluents of these areas.

## 1. Introduction

One of the highest public health challenges worldwide is the increase in the number and types of antimicrobial resistances (AMR) [1]. The use and misuse of antimicrobials in human medicine is one of the main causes of this increasing problem, but inappropriate practices in intensive livestock farms have also contributed to the alarming increase in antibiotic resistant bacteria (ARB) [2,3]. Antibiotics used in animal production with different purposes (therapeutically and prophylactically) are finally disseminated through the environment. It has been estimated that about 75% of the administered antibiotics is not absorbed by animals and is excreted via the feces or urine [4]. In this sense, García-Galán et al., 2011 [5] reported the presence of emerging pollutants in the Ebro basin (area with intensive livestock farms), including at least eight types of antibiotics. As a consequence of the antibiotic pressure, ARB have been isolated from different sources such as farms (manure), water and meat products [4,6,7,8]. The dissemination of AMR throughout the environment represents a risk to human health [9,10]. In particular, one of the main routes for the dissemination of ARB and resistance genes (ARGs) is the aquatic environment [11,12]. Therefore, a One Health approach is needed to address the problem of antimicrobial resistance. Recently, the World Health Organization (WHO) has published a list of antibiotic-resistant priority pathogens with the aim to promote research and development of new antibiotics, as one of the proposed strategies to control the problem of global resistance to antimicrobial medicines [13]. The list includes 12 families of bacteria that pose the greatest threat to human health, especially if they are spread throughout the environment. Among pathogens classified as priority 1 (critical) and 2 (high), carbapenem, β-lactam, vancomycin and methicillin resistances are considered. β-lactam antibiotics have been the most extended therapeutic choice for the treatment of human and animal infections worldwide, and consequently, bacteria have developed different β-lactam resistance mechanisms, such as the production of extended spectrum β-lactamases (ESBLs) and carbapenemases [14]. The presence of *bla* genes encoding SHV, TEM, CTX-M groups, KPC, NDM and VIM enzymes has been frequently reported in rivers of different regions over the world [15,16]. Despite methicillin-resistant *Staphylococcus aureus* (MRSA) has been detected basically in clinical environments [17,18], the presence of MRSA *mecA/C* in river water has been described [19]. Regarding vancomycin resistant enterococci (VRE), although the presence of these bacteria seems to be related to small wild mammals, rabbits and birds [20,21], some authors described the presence of *E. faecium vanA* and *vanB* in wastewater and surface waters [22,23]. Colistin has become as the last alternative in human medicine for the treatment of infections due to multidrug-resistant Gram-negative bacteria [24]. Colistin sulfate is used for the control of Enterobacteriaceae infections in pig production in some countries [25,26], contributing to the spread of colistin resistances mediated by the transferable plasmid *mcr-1* [27]. In this context, it would be very interesting to search for this type of resistance in different aquatic environments, such as rivers, WWTPs and collectors.

POCTEFA 2014-2020 is the acronym for the INTERREG V-A Spain-France-Andorra Program (https://www.poctefa.eu/). It is a European territorial cooperation program created to promote the sustainable development of the regions near to the Franco-Spanish border (Navarra, Huesca, Zaragoza, Lleida, Pyrénées-Atlantiques, Hautes-Pyrénées, Orientals-Pyrénées, Haute Garonne and Ariege). This area covers a region of 115.583 km^2^, populated by 15 million habitants, being the intensive livestock farms as the main rural economic engine (especially porcine, poultry and rabbit production). In this sense, the main objective of this study was to determine the presence of ARB in rivers, wastewater treatment plants (WWTPs) and collectors in the North of Spain and South of France (hereafter named POCTEFA area). Specifically, we focused the study on the isolation and characterization of critical and high priority resistant pathogens according to the WHO list: Enterobacteriaceae, *Pseudomonas aeruginosa* and *Acinetobacter baumanii* carbapenem-resistant; Enterobacteriaceae ESBL-producing; *Enterococcus faecium* vancomycin-resistant and *Staphylococcus aureus* methicillin-resistant. In addition, due to the recent interest in colistin resistances, we also included the search for Enterobacteriaceae colistin-resistant.

## 2. Materials and Methods

### 2.1. Sample Collection

**Figure 1:**
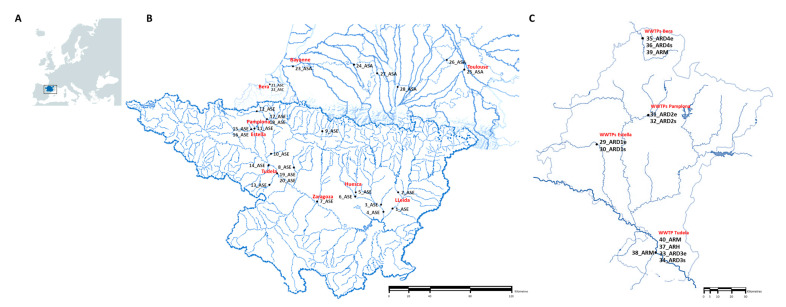
Geographical location of sampling. (**A**) POCTEFA area (North Spain and South France). (**B**) Sampling points of rivers (ASE: Ebro Basin; ASC: Cantabrian Basin; ASA: Adour-Garonne Basin); (**C**) Sampling points of wastewater treatment plants (WWTPs) and collectors in the Navarra region (ARDe: Discharge of wastewater entering the treatment plant. ARDs: Discharge of wastewater leaving the treatment plant. ARH: Discharge of hospital wastewater. ARM: Discharge of slaughterhouse wastewater).

The sampling was performed by trained people from the University of Zaragoza (Laboratory of Water and Environmental Health) in 40 locations from POCTEFA area including rivers, WWTPs and collectors (hospital and slaughterhouses). A total of 80 samples were collected in two seasons of 2018 (April–May and October–November). Rivers were located in France and Spain, whereas WWTPs and collectors were present in Navarra (Northern Spain; Figure 1). Sampling of WWTPs was performed in influent and effluent water and sampling of rivers was done upstream and downstream of the WWTP (when present). Complete information of each point, provided by the Laboratory of Water and Environmental Health of University of Zaragoza, is available in the supplementary material (Tables S1 and S2).

Samples were taken in sterile containers in accordance with ISO 19458 [28] and ISO 5667-3 [29] standards and stored at 5 ± 3 °C in the dark until they were sent to the University of Navarra. Microbiological analysis was carried out within 24 h of arrival of samples (stored at 5 ± 3 °C).

### 2.2. Isolation and Identification of Resistant Bacteria

In order to detect the presence of resistant bacteria (even the lethargic ones by environmental stressors such as the temperature or the lack of nutrients), two approaches were performed. In the first method, 1 mL of each sample was spread on the surface of specific selective culture media for each resistance type (described below). In the second method, two previous enrichment processes were carried out for the recovery of stressed cells. This way, 10 mL water samples were transferred to 10 mL of double concentration EE Mossel broth (Difco, Le Pont de Claix, France) and were incubated at 37 ± 1 °C during 24 h, in order to isolate Gram negative bacteria. Similarly, enrichment in Giolitti Cantoni broth (Oxoid, Basingstoke, United Kingdom) was performed for the recovery of Gram positive bacteria (24–48 h at 37 ± 1 °C). Following the incubation periods, isolations were performed on the selected culture media. In the case of carbapenem and colistin resistances, selective culture media was changed in the second sampling in order to improve the recovery of these strains (taking into account the obtained results in the first sampling).

Chromogenic selective plates from bioMerieux (Marcy l’Etoile, France) were used for the isolation of the target resistant bacteria. Thus, ChromID ESBL plates containing a mixture of antibiotics including cefpodoxime (CPD) were used for the isolation of suspicious ESBL-producing strains. ChromID MRSA contains cefoxitin (FOX) as a selective agent and was used for the isolation of MRSA. ChromID VRE agar plates selects vancomycin (VA) resistant *Enterococcus*, allowing the differentiation between *E. faecium* and *E. faecalis*. Finally, ChromID CARBA SMART agar plates and ChromID CARBA agar plates were used for the isolation of carbapenemase-producing Enterobacteriaceae (CPE; first and second sampling events, respectively). In addition, Columbia CNA and MacConkey agar supplemented with 2 µg/mL of colistin (COL; Oxoid) were used for the isolation of colistin resistant bacteria (first and second sampling events, respectively). After the incubation at 37 ± 1 °C during 24–48 h, suspicious colonies were isolated on ChromID CPSE, nutrient agar or blood agar (bioMerieux). The identification was carried out using matrix-assisted laser desorption/ionization time-of-flight mass spectrometry (MALDI-TOF-MS; bioMerieux) or biochemical tests (oxidase, API 20NE, API20E, APIstaph or API 20STREP; bioMerieux). Pure cultures were stored at −80 °C for further characterization.

### 2.3. Phenotypic and Genotypic Characterization of Resistant Strains

The antibiotic disks used for the phenotypic characterization were provided by Becton Dickinson (Le Pont de Claix, France), ROSCO Diagnostica (Taastrup, Denmark) and by Biomerieux in the case of the E-tests. The results were interpreted according to “Clinical & Laboratory Standars Institute”, CLSI [30] or “The European Committee on Antimicrobial Susceptibility Testing”, EUCAST guidelines [31]. The antimicrobials tested and resistance breakpoints can be found in the supplementary material (Table S3). The specific methodology applied for the phenotypic and/or genotypic characterization of each types of resistance is explained in the subsequent sections.

#### 2.3.1. ESBL-Producer Enterobacteriaceae and Other β-Lactamases

ESBL production was confirmed by the double-disk synergy test (DDST) according to Jarlier et al., 1988 [32]. Basically, the amoxicillin/clavulanic acid (AMC, 30 μg) was placed in the center of the inoculated Mueller Hinton cation-adjusted agar plate (MH; Becton Dickinson) and the following β-lactam antibiotics were placed at a distance of 20 mm: ceftazidime (CAZ, 30 μg), ceftriaxone (CRO, 30 μg), aztreonam (AZT, 30 μg) and cefpodoxime (CPD, 10 μg). After incubation at 37 ± 1 °C for 18–24 h, the strain was considered as the ESBL-producer when the enhanced inhibition zone was observed between the cephalosporin disk and AMC, indicating synergy. AmpC β-lactamase production was determined following the methodology of Thean et al., 2009 [33] by comparing the diameters of each β-lactam or β-lactam with an inhibitor (ceftazidime/clavulanic acid and cefotaxime/clavulanic acid) in MH and MH supplemented with cloxacillin (250 mg/L, Sigma Aldrich, Singapore). When an increased inhibition zone of >5 mm in cloxacillin plates was observed, the microorganisms was considered to be an AmpC-producer [34]. Finally, we studied the presence of metallo-β-lactamases (MBL) according to Arakawa et al., 2000 [35], using CAZ (30 μg), imipenem (IMP, 10 μg) and EDTA (10 μL) disks in MH plates. In addition, an IMP disk was used to which 10 µL of EDTA was added. It was considered an MBL-producing strain when a synergistic effect was observed between the IMP, CAZ and EDTA discs and if the difference between the IMP + EDTA disc and the IMP disc was >5 mm.

The DNA extraction procedure was performed with the DNeasy^®^ Blood and Tissue kit (Qiagen, Barcelona, Spain), using a pretreatment protocol for Gram-negative bacteria and following the manufacturer’s instruction. The quantity and quality of the DNA was analyzed using a Nanodrop ND-1000 spectrophotometer (NanoDrop Technologies, Wilmington, DE, USA).

The detection of AmpC β-lactamases genes was performed using the multiplex-PCR assay described by Pérez-Pérez and Hanson, 2002 [36]. The primers, size of the amplicons and conditions followed are summarized in Table 1.

The identification of *bla*_TEM_, *bla*_SHV_ and *bla*_OXA_ genes was performed using the multiplex-PCR assay described by Colom et al., 2003 [37] while a modification of the multiplex-PCR described by Woodford et al., 2006 [38] was used for the study of *bla*_CTX-M_ genes. The reaction mixture composition and amplification conditions for the *bla*_CTX-M_ genes were described in the manuscript of Ojer-Usoz et al., 2014 [8]. All the details for the several multiplex PCR assays are shown in Table 1.

A bidirectional DNA sequence analysis of the amplicons were performed by the Macrogen EZ-Seq purification service to determine the molecular types of *bla* genes (Macrogen Europe, Amsterdam, The Netherlands). Searches for DNA and protein homologies were carried out through the National Centre for Biotechnology Information (http://www.ncbi.nlm.nih.gov/) using the BLAST program. The alignment of DNA and amino acids sequences was performed using Clustal Omega (http://www.ebi.ac.uk/Tools/msa/clustalo/).

#### 2.3.2. Carbapenemase-Producing Strains

Disks of ertapenem (ETP, 10 μg, Oxoid), IMP (10 μg, Oxoid) and meropenem (MER, 10 μg, Becton Dickinson) were used to determine carbapenemase production. In addition to the screening of the presence of β-lactamase and metalo-β-lactamase (described above), OXA-48-like, KPC, NDM and VIM type carbapenemases were determined using the immunochromatography test Resist-4 O.K.N (Coris Bioconcept, Gembloux, Belgium), according to the manufacturer instructions.

#### 2.3.3. Colistin Resistant Enterobacteriaceae

COL E-test (Biomerieux) was performed for the determination of this resistance, using EUCAST guidelines [31] for the interpretation of the inhibition zone (Table S3). The presence of *mcr-1*, *mcr-2*, *mcr-3*, *mcr-4* and *mcr-5* genes was detected by conventional PCRs using the specific primers and conditions shown in Table 2 and following the conditions described in the corresponding works [39,40,41,42,43].

#### 2.3.4. Methicillin Resistant Strains

Methicillin resistances were confirmed by using FOX disks (30 μg, Becton Dickinson). For the determination of gene *mecA*, the Alere^TM^ PBP2a test was performed according to the manufacturer instructions (Abbot, Scarborough, Maine). This is a rapid qualitative immunochromatographic analysis for the detection of penicillin 2a binding protein (encoded by *mecA*).

#### 2.3.5. Vancomycin Resistant Strains

The VA resistance of *E. faecium* was confirmed by the E-test (bioMerieux), in Mueller Hinton Agar with 5% sheep blood (bioMerieux). Additional E-test of teicoplanin (TEC; bioMerieux) was performed in positive strains in order to determine the presence of gene *vanA* or *vanB*. Strains with the *vanA* phenotype are characterized by a high level of resistance to both VA (MIC ≥ 64 µg/mL) and TEC (MIC ≥ 16 µg/mL). However, strains carrying the *vanB* gene are characterized by variable levels of resistance to VA (MIC between 4 and ≥ 1000 µg/mL) and sensitivity to TEC [20].

### 2.4. Antimicrobial Resistance Patterns

The antimicrobial susceptibility of resistant strains to additional antibiotics was obtained in the MicroScan^®^ system (Siemens AG, Munich, Germany). NM37, PN28 and Neg Multidrug Resistant MIC 1 panels (Siemens AG, Germany) were used in combination with Lab Pro^®^ 3.5 software for determining the minimum inhibitory concentrations (MICs). The panels included the following antimicrobials: AMC, ampicillin (AMP), ampicillin-sulbactam (AMS), azithromycin (AZI), AZT, cefazolin (CZ), cefepime (FEP), CAZ, cefuroxime (CXM), CPD, cefotaxime (CTX), FOX, chloramphenicol (CHL), ciprofloxacin (CIP), COL, clindamycin (Cd), daptomycin (DAP), ETP, erythromycin (ERY), fosfomycin (FOT), fusidic acid (FA), gentamicin (GM), IMP, levofloxacin (LV), linezolid (Lz), MER, mupirocin (MUP), moxifloxacin (MXF), mezlocillin (MZ), norfloxacin (NOR), nitrofurantoin (FD), oxacillin (OX), penicillin (P), piperacillin (PIP), piperacillin-tazobactam (TZP), rifampicin (RA), synercid (SYN), tobramycin (TO), tetracycline (TET), tigecycline (TIG), TEC, trimethoprim-sulfamethoxazole (SXT) and VA.

This automated method provided very interesting results for the study of ESBL-producing bacteria. ESBL production was confirmed when a > 3 two-fold concentration decrease occurred in an MIC for any of β-lactams tested in combination with clavulanic acid versus its MIC when tested alone [8,44]. The MIC50 and MIC90 (minimum concentration required to inhibit the growth of 50% and 90% of organisms, respectively) were used to evaluate antibiotic sensitivities. Multi-drug resistances (MDR) and extensive MDR were considered when resistances to three or at least five antimicrobial agents were detected, respectively [45].

### 2.5. Statistical Analysis

The results for the rates of resistances to antibiotics were subjected to statistical processing with the SPSS 15 software (SPSS Inc., Chicago, IL, USA), applying the Chi-square (X^2^) test with a level of significance of *p* < 0.05.

## 3. Results

### 3.1. Prevalence of Resistant Bacteria in Rivers and Sewage Waters

Table 3 shows the positive samples (in red) regarding antibiotic resistances in both sampling events. In relation to rivers (samples 1–28), 96.4% were carriers of antibiotic resistant bacteria for at least one of the selected family types, being only one river in the Ebro basin (9_ASE) negative in both samplings. However, a lower prevalence of positive samples was detected in the Adour-Garonne Basin (samples 23 to 28), and COL resistances were not detected in the French area.

As shown in Table 4, the prevalence was similar in both sampling events (with the exception of carbapenem and COL, *p* < 0.05), the resistances to CPD and FOX being the prevalent ones. Changes in the methodology for a better isolation of COL and carbapenem resistant bacteria may be the cause of the increase of positive samples for these antibiotics in the second sampling.

Regarding WWTP and collectors, 100% of the samples were positive for at least one type of resistance in both samplings and no differences were observed (*p* > 0.05) between the influent and effluent water in the treatment plants (Table 3). It must be noticed that the greatest variety and number of target resistant bacteria were isolated from wastewaters (*n* = 32, 28.9%). In this way, all target carbapenem resistant pathogens (*n* = 7) and the majority (80%) of *E. faecium* resistant to VA were isolated from samples of influent water of sewage treatment plants and collectors, while no MRSA was detected (Table 4).

**Figure 2:**
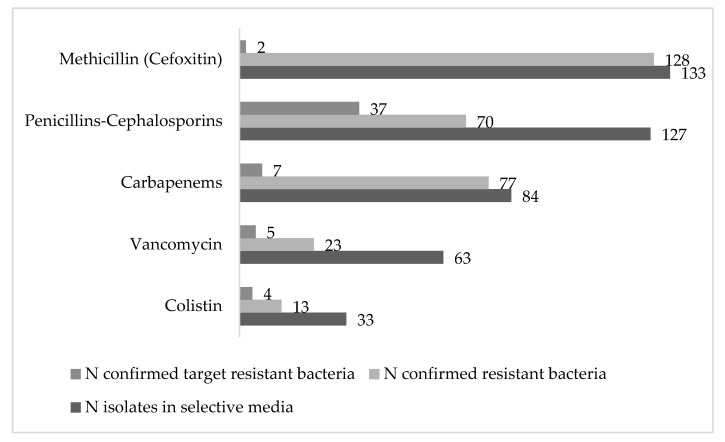
Total number of resistant strains isolated from water samples in the POCTEFA area.

From the total of 440 strains isolated in selective media, 311 (70.7%) were confirmed as resistant by the aforementioned phenotypic methods (Figure 2).

**Figure 3:**
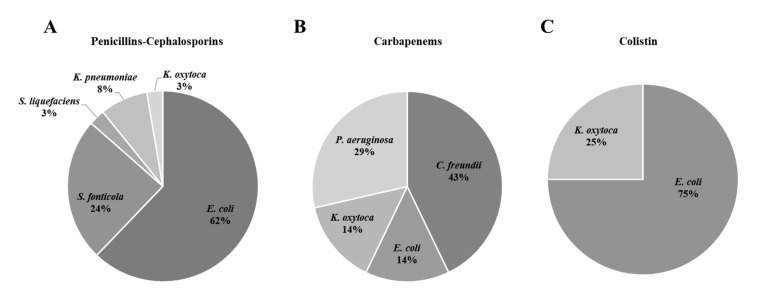
Genus distribution of isolated Gram negative target resistant bacteria. (**A**) Penicillins-Cephalosporins; (**B**) Carbapenems and (**C**) Colistin.

In general, penicillin and cephalosporin resistances were the most extended ones (63.7%), followed by carbapenem resistances (24.7%) and a lower prevalence of VA and COL resistances was detected (7.4% and 4.2%, respectively). Due to the large number of environmental strains isolated with innate resistance to these groups of antibiotics (mainly penicillin resistance), only 17.7% (*n* = 55) of the isolates were identified as target priority pathogens (with acquired resistance), with a high presence of ESBL-producers (52.8% *n* = 37). The majority of isolates of this target group were identified as *E. coli* (62%) followed by *Serratia* (Figure 3A). Similarly, only 2 out of 128 isolates resistant to FOX (1.6%) were confirmed as MRSA. Regarding carbapenemase-producing isolates 7 out of 77 (9%) corresponded to the list of priority pathogens (*P. aeruginosa* and Enterobacteriaceae) and no *A. baumannii* was isolated (Figure 3B). In addition, 4 out of the 13 COL resistant Enterobacteriaceae (30.8%) were identified, *E. coli* being the prevalent one (Figure 3C). Finally, five *E. faecium* (21.7%) were identified from the total of 23 VA resistant isolates.

### 3.2. Characteristics of the Target Isolated Pathogens

A summary of the antimicrobial resistance patterns and antimicrobial resistance genes (ARG) of the 48 target Gram negative isolates is present in Table 5.

**Figure 4:**
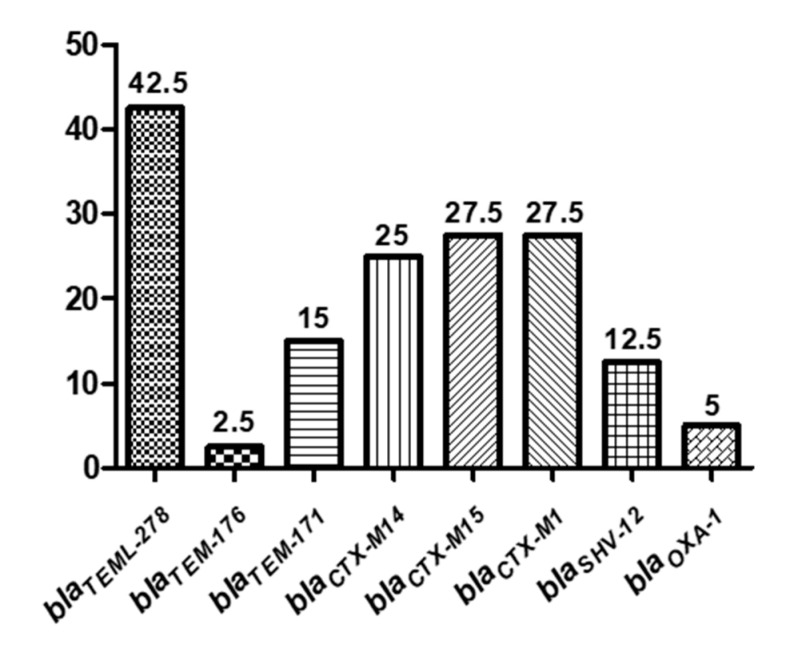
Prevalence (percentage) of β-lactamase genes in Gram negative isolates.

ESBL production was confirmed by the double-disk synergy test (DDST) and MicroScan^®^ system in a 93.7% (*n* = 45) of the strains. However, the presence of *bla* genes was confirmed by PCR and sequencing in 88.9% (*n* = 40) of them. This could be probably related with the higher specificity of these genotypic methods in *E. coli* strains rather than in other species (*Serratia*, *Klebsiella* or *Pseudomonas*). Regarding the incidence of *bla* genes, the prevalent one was *bla*_CTX-M_ (80%), followed by *bla*_TEM_ (60%), *bla*_SHV-12_ (12.5%) and *bla*_OXA-1_ (5%; Figure 4). The sequence analysis demonstrated that genes from CTX-M1 group (*bla*_CTX-M1_ and *bla*_CTX-M15_) were present in 55% of ESBL-producing isolates (Figure 4). Regarding the CTX-M-9 group, it was present in 25% of ESBL-producing isolates and the sequences shown that all of them corresponded to the *bla*_CTX-M-14_ gene. Genes encoding for *bla*_CTX-M9_, *bla*_CTX-M2_, *bla*_CTX-M8_ and *bla*_CTX-M25_ were not detected. Sequence analysis demonstrates that all isolates carrying the β-lactamase TEM (Temoneira) belonged to the class TEM-1, being *bla*_TEML-278_ the prevalent one. In general, CTX-M and TEM *bla* genes were widely distributed among all the water sources, but *bla*_SHV-12_ and *bla*_OXA-1_ were mainly detected in WWTPs plants. Furthermore, 18 (45%) of the isolates had two or more *bla* genes (Table 5). Specifically, one of the *K. pneumoniae* strains carried four different *bla* types (*bla*_TEML-278_, *bla*_SHV-12_, *bla*_OXA-1_ and *bla*_CTX-M15_). In addition, *bla*_OXA-1_ and *bla*_SHV-12_ genes were always detected together with other β-lactamase genes.

Otherwise, the presence of metallo-β-lactamases was not observed in any of the selected strains, while 50% of isolates (*n* = 24) were confirmed as AmpC-β-lactamases-producers (Table 5). Nevertheless, AmpC genes (ACC, DHA and EBC) were detected in only five strains (numbers 30, 32, 34, 41 and 42 in Table 5). With the exception of the river isolate (number 30), the remaining four strains came from a WWTP and a duck slaughterhouse collector located in the same geographical area. Furthermore, all the strains had been characterized as carriers of other types of *bla* genes. In particular, the *K. pneumoniae* isolate number 34, contained three types of *bla* genes (*bla*_TEM-171_, *bla*_SHV-12_ and *bla*_CTX-M1_). Regarding carbapenemase-producing isolates, all of them were negative for carbapenemases type OXA-48, NDM and VIM. However, the presence of KPC-type carbapenemases was confirmed in four strains (36.3%), corresponding with the strains isolated from the aforementioned WWTPs and the duck slaughterhouse collector (numbers 38, 39, 41 and 42), in which AmpC genes were detected (Table 5). Furthermore, all the carbapenemase-producing bacteria were ESBL carriers, *bla*_TEML-278_ being the most extended *bla* gen (71.4%). In another way, despite all Enterobacteriaceae being confirmed as COL resistant by the E-test and MicroScan^®^ system, the presence of the phosphoethanolamine transferase *mcr-1* gene was confirmed in only one *E. coli* (number 47 in Table 5) and all the strains were negative for the rest of the variants of *mcr*. This *E. coli mcr-1* was isolated from the collector of a rabbit slaughterhouse and was resistant to quinolones, tetracyclines and SXT, while it was not resistant to β-lactam antibiotics (Table 5). In fact, from the four COL-resistant isolates, only one strain of *E. coli* isolated from duck slaughterhouse collector (number 32) was resistant to this group of antibiotics and carried the *bla*_CTX-M15_ gene

With respect to the target Gram positive resistant isolates (*n* = 7, Table 6), the five strains identified as VA resistant *E. faecium* (VRE) were sensitive for TEC and considered as the *vanB* phenotype according to the recommendations (CLSI, 2018). Finally, the two MRSA isolates were negative in the Alere^TM^ PBP2a test for checking the presence of the *mecA* gene, the most widespread genetic mechanism involved in this resistance.

### 3.3. Multidrug Resistance Profiles

MDR (resistance to at least three antibiotics families) and extensive MDR (at least to five families) were observed in 96.4% and 67.2% of the strains respectively, with MDR being observed in 100% of the Gram positive strains (Table 5 and Table 6). High levels of resistance against penicillins, cephalosporins and β-lactamase inhibitors were detected in the Gram negative isolates (*n* = 48). In fact, the higher resistances were observed in AMP (95.8%), CZ (93.75%), CXM (91.6%), CPD (91.6%) and CTX (89.6%), followed by FEP (70.8%) and CAZ (66.6%). Furthermore, the majority of the isolates (77%) showed susceptibility to carbapenems (ETP, MER and IMP), despite the fact that some isolates showed MIC values in the MicroScan^®^ close to the breaking point for IMP. In relation to monobactams, the 68.7% of strains were resistant against AZT, while more reduced resistance against tetracyclines (41.6%), CHL (29.2%) and aminoglycosides (25%) was observed. The percentage of resistance against quinolones and sulfonamides was approximately 50%. Finally, resistance to COL was the least prevalent (10.4%), with only five positive confirmed strains.

Regarding Gram positive strains (*n* = 7), 100% of isolates were resistant to ERY and the majority (85.7%) were resistant to OX and fourth generation cephalosporins (FEP). Furthermore, resistance to glycopeptides, such as VA, was also prevalent (85.7%). In addition, one of the MRSA isolates was also resistant to VA and TEC, an important fact since MRSA with intermediate resistance to VA are also considered in the WHO list. Likewise, 71.4% of isolates were resistant to carbapenems, whereas the resistance against aminoglycosides (57.1% GM and 28.6% TO), tetracyclines (57.1%) and quinolones (42.9%) was lower.

**Figure 5:**
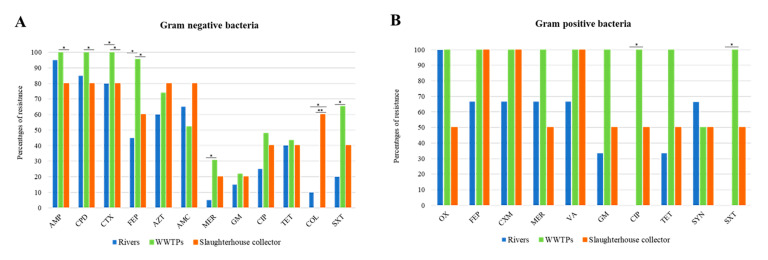
Resistance rate to each antibiotic according to the isolates origin. (**A**) Gram negative strains and (**B**) Gram positive strains. AMP (ampicillin); CPD (cefpodoxime); CTX (cefotaxime); FEP (cefepime); AZT (aztreonam); AMC (amoxicillin-clavulanic acid); MER (meropenem); GM (gentamicin); CIP (ciprofloxacin); TET (tetracycline); COL (colistin); SXT (trimethoprim-sulfamethoxazole); OX (oxacilin); CXM (cefuroxime); VA (vancomycin); SYN (synercid). (*, *p* < 0.05, **, *p* < 0.01).

The resistance rates to each individual antibiotic according to the isolates origin is represented in Figure 5A,B, for Gram negative and Gram positive strains, respectively. Regarding Gram negative bacteria, strains isolated from wastewater had the highest resistance rates for AMP, CPD, CTX, FEP, MER and SXT (*p* < 0.05). Besides, significant differences were found between rivers and WWTPs for COL resistance (*p* < 0.05). In fact, very significant differences were found among WWTPs and collectors (*p* = 0.0001). Concerning Gram positive bacteria, only significant differences were found between rivers and WWTPs for CIP and SXT (*p* < 0.05).

## 4. Discussion

This study aimed to determine the prevalence of antibiotic resistant bacteria in aquatic environments of the POCTEFA area, a region of intensive livestock activity. The widespread presence of resistant bacteria observed in rivers, WWTPs and collectors (96.4% and 100%, respectively), highlight the impact of human activity on the spread of these resistances, especially from hospital and livestock production. In fact, 55 resistant strains identified as critical and high priority resistant pathogens (according to WHO list) were isolated in the study.

The wastewater from slaughterhouses is considered a relevant source of antimicrobial resistant bacteria and consequently may be important for its diffusion into the environment [46]. The livestock pressure in most of the Spanish rivers studied in this work was high, where pig farms stood out mainly (Table S1). This could be the reason of the higher prevalence of resistances in the Spanish rivers than the French ones (Table 3). In addition, the unique negative river regarding the presence of resistant bacteria (9_ASE) was located in the Pyrenees area, where no relevant cattle exploitations such as pigs, birds and rabbits were reported.

In agreement with our results, previous studies reported an increase of ESBL and carbapenemase-producing Enterobacteriaceae (CPE) in rivers and WWTPs, with high percentages of clinically relevant multidrug resistant bacteria and related genes (*intI*1, *sul*1, *bla*_OXA_, *mcr-1*, *bla*_CTX-M15_, *bla*_KPC_ and *bla*_VIM_, among others) that were still present in effluent samples, indicating an insufficient reduction during conventional wastewater treatment [47]. In this line, our results are in agreement with previous work published by Ojer-Usoz in the same region of Navarra [8], with a similar prevalence of ESBL after 6 years. In addition, the association between *bla*_OXA-1_ and resistance to aminoglycosides and quinolones reported by Osińska et al., 2016 [48] was confirmed in the present study, because the two WWTP isolates carrying *bla*_OXA-1_ were resistant to TO, LV, CIP, MXF and NOR. Indeed, the increased quinolone resistance rate in the isolates (50%) may be caused by the use of enrofloxacin in slaughtered broiler herds [46]. Furthermore, the high prevalence regarding AmpC β-lactamases could be related with the large number of *Serratia* and *Citrobacter* strains (carriers of chromosomal AmpC). In addition, it is interesting to keep in mind that ESBL and AmpC coproduction was detected in 19 strains, despite only five strains being confirmed as AmpC-producers by molecular methods. Moreover, two of them (numbers 39 and 42 in Table 5), were also carriers of carbapenemase gene KPC. The isolation of different KPC producing species (*E. coli*, *K. oxytoca* and *C. freundii*) in the same water sample (33_ARD3e) reinforced the hypothesis that a horizontal gene transfer is taking place between different bacterial species. Finally, in this study we did not isolate *A. baumanii* resistant to penicillins, cephalosporins or carbapenems. This could be related to the low presence of this pathogen or with methodological problems on the isolation of this species.

Colistin (polymyxin E) is currently used as a last alternative drug against MDR Gram negative bacteria. However, its resistance has even emerged in humans since it has been widely used in pig production and in some countries in veterinary (especially in cows) for the treatment of gastrointestinal infections caused by Enterobacteriaceae [26]. This resistance is frequently related to chromosomal mutations, nonetheless, the mechanism by which the *mcr-1* gene confers resistance to COL was the first one that described plasmid mediated transmission antibiotic resistance and was first discovered in China on a pig farm [39]. Despite this gene being widespread in the environment [49] and it having been documented in 30 strains isolated from three Spanish WWTPs [50], only one *E. coli* strain isolated from a rabbit slaughterhouse collector (number 47 in Table 5) was positive for *mcr-1* in our study.

Enterococci are recognized as important nosocomial pathogens due to their natural intrinsic resistance and their ability to acquire resistance to multiple drugs [49]. The resistance to VA in enterococci (VRE) is associated with the use of this antibiotic in clinics and, as a consequence, effluents from hospitals constitute an important point for the transmission of this resistance [51]. In this sense, despite the fact that VA resistant bacteria were isolated from the hospital collector (37_ARH), none of these isolates was identified as VRE. However, VRE were present in the influent waters of the WWTPs of two points near hospitals (29_ARD1e and 33_ARD3e), in accordance with other studies [52]. In general, VA resistances are specially linked to *vanA* and *vanB* genes and represent a major public health problem, due to their resistant gene transfer capacity [53]. In this sense, *vanB* carriers are characterized by high levels of VA resistance and TEC sensitivity, and the resistance is transferred by conjugation associated with the mobilization of genetic material through the acquisition and/or exchange of transposons [20]. In agreement with that, all our VRE isolates showed the *vanB* phenotype. As VA is not used in veterinary medicine, the use of other glycopeptides as an animal growth promoter (such as avoparcin), was associated with the increase in VRE in the 70s [20]. Numerous studies have shown that VRE persisted in animals for a long time after avoparcin was banned [49]. Therefore, the presence of identical resistance genes in animal and human enterococci, suggest the spread between isolates from different environments [53]. The isolation of *E. faecium* VA resistance in samples from a rabbit slaughterhouse collector (40_ARM) reinforces this hypothesis. Finally, it is known that VRE can rapidly develop resistance after the introduction of new antimicrobial agents in the clinic, such as quinupristin-dalfopristin (SYN), Lz and DAP [54]. So, it should be noted that 80% of the *E. faecium vanB* isolates of this study were resistant to SYN and 40% were resistant to DAP, whereas no resistances to Lz were observed.

One of the most important acquired resistances in *S. aureus* is methicillin resistance (MRSA) and is mainly due to the acquisition of the *mecA* gene, encoding a β-lactam low affinity penicillin binding protein (PBP) called PBP2a [49]. In general MRSA isolates from surface water are quite rare, with only a low number of isolates [51]. Despite this, MRSA *mecA* has been reported to survive in rivers and municipal wastewater and had been associated with colonized people [19]. In addition, the presence of gene *mecC* has been reported for the first time in a Spanish river, highlighting the potential role of water in the dissemination of *mecC* MRSA [19]. *S. aureus mecC* was also isolated from animals and an urban wastewater treatment plant [55] and other studies highlighted the emergence of *S. aureus mecC* in livestock production, particularly in pigs in European countries [56]. In this sense, our two MRSA strains (negative for the *mecA* gene in the PBP2a test) were isolated from rivers (numbers 54 and 55 in Table 6) with high incidence from pig exploitations, which would reinforce this hypothesis.

In general, the main objective of wastewater treatment is to eliminate organic (chemical and biological) components, phosphorous and nitrogen nutrients as well as suspended solids. Directive 91/271/EEC [57] establishes the guidelines to be followed by the Member States of the European Union to ensure that urban wastewater receives adequate treatment before discharge, but it does not include disinfection processes that reduce the microbiological charge and ARGs in the effluents [58]. Consequently, these bacteria are incorporated into the environment through the direct or indirect discharge of treated water or through sludge, which finally is used as a fertilizer in agricultural practices. In the same way, the directive does not provide specific restrictions for effluents from hospital wastewater, which also constitute an important reservoir of ARB [52]. It is known that some ARB can be removed through conventional wastewater treatment processes [6], but still large numbers that survive in the effluent. Therefore, tertiary treatment methods or advanced treatment technologies are those that manage to eliminate some bacterial load and genes [47]. In this sense, UV and ozone-treatment have been investigated for a long time with the aim of reducing these microbial loads. UV disinfection contributes to the effective reduction of some bacteria, like 99.9% of MRSA or VRE [6]. However, Munir et al., 2011 [59] founded that this disinfection did not contribute to the reduction of TET and sulfonamide resistant bacteria. Moreover, ozonation is an efficient process to eliminate organic microcontaminants and for inactivating bacteria through the production of highly reactive radical [60]. Other tertiary treatments are based on the water exposure to solar radiation in the lagoon and according to López Martínez [61] are able to reduce the microbiological concentration up to four orders of magnitude at the longest time of exposure to solar radiation. However, these advanced wastewater treatment technologies are also known to accelerate horizontal gene transfer due to the activation of different repair mechanisms involved in the dissemination of antibiotic resistance genes [6]. Consequently, it is necessary to develop other additional strategies and guidelines for the elimination of microbial contaminants in wastewater, which included surveillance of pathogenic bacteria and ARGs. For that reason, there is a need to improve effective disinfection measures and treatments in WWPTs and animal slaughterhouses to avoid environmental contamination and prevent the evolution of antibiotic resistance.

## 5. Conclusions

The results of this study highlight that resistant bacteria to clinically relevant antibiotics were present in the different water samples examined in the POCTEFA area, with a higher presence in wastewaters from slaughterhouses, hospitals and WWTPs. In order to minimize the dispersion of resistances through the effluents of these areas, it is necessary to implement effective methods of wastewater disinfection and surveillance programs of ARB.

## Figures and Tables

**Table 1:** Primers and conditions used for the amplification of the different β-lactamases genes.

Target	Primer	Sequence (5′–3′)	Amplicon Size (bp)	Conditions
*bla*_MOXM_	*bla*_MOXM_-Fw	GCTGCTCAAGGAGCACAGGAT	520	Initial denaturation at 94 °C for 3 min; 25 cycles of amplification: denaturation at 94 °C for 30 s, hybridization at 64 °C for 30 s and extension at 72 °C for 1 min; final elongation at 72 °C for 7 min.
	*bla*_MOXM_-Rv	CACATTGACATAGGTGTGGTGC		
*bla*_CITM_	*bla*_CITM_-Fw	TGGCCAGAACTGACAGGCAAA	462	
	*bla*_CITM_-Rv	TTTCTCCTGAACGTGGCTGGC		
*bla*_DHAM_	*bla*_DHAM_-Fw	AACTTTCACAGGTGTGCTGGGT	405	
	*bla*_DHAM_-Rv	CCGTACGCATACTGGCTTTGC		
*bla*_ACCM_	*bla*_ACCM_-Fw	AACAGCCTCAGCAGCCGGTTA	346	
	*bla*_ACCM_-Rv	TTCGCCGCAATCATCCCTAGC		
*bla*_EBC_	*bla*_EBC_-Fw	TCGGTAAAGCCGATGTTGCGG	302	
	*bla*_EBC_-Rv	CTTCCACTGCGGCTGCCAGTT		
*bla*_FOX_	*bla*_FOX_-Fw	AACATGGGGTATCAGGGAGATG	190	
	*bla*_FOX_-Rv	CAAAGCGCGTAACCGGATTGG		
*bla*_SHV_	*bla_SHV_*-Fw	AGGATTGACTGCCTTTTTG	392	Initial denaturation at 94 °C for 5 min; 32 cycles of amplification: denaturation at 94 °C for 30 s, hybridization at 54 °C for 30 s and extension at 72 °C for 1 min; final elongation at 72 °C for 10 min.
	*bla*_SHV_-Rv	ATTTGCTGATTTCGCTCG		
*bla*_TEM_	*bla*_TEM_-Fw	ATCAGCAATAAACCAGC	516	
	*bla_TEM_*-Rv	CCCCGAAGAACGTTTTC		
*bla*_OXA_	*bla*_OXA_-Fw	ATATCTCTACTGTTGCATCTCC	619	
	*bla*_OXA_-Rv	AAACCCTTCAAACCATCC		
*bla*_CTX-M1_	*bla*_CTX-M1_-Fw	AAAAATCACTGGCCAGTTC	415	Initial denaturation at 94 °C for 5 min; 30 cycles of amplification: denaturation at 94 °C for 45 s, hybridization at 55 °C for 30 s and extension at 72 °C for 1 min; final elongation at 72 °C for 6 min.
	*bla*_CTX-M1_-Rv	AGCTTATTCATCGCCACGTT		
*bla*_CTX-M2_	*bla*_CTX-M2_-Fw	CGACGCTACCCCTGCTATT	552	
	*bla*_CTX-M2_-Rv	CCAGCGTCAGATTTTTCAGG		
*bla*_CTX-M9_	*bla*_CTX-M9_-Fw	CAAAGAGAGTGCAACGGATG	205	
	*bla*_CTX-M9_-Rv	ATTGGAAAGCGTTCATCACC		
*bla*_CTX-M8_	*bla*_CTX-M8_-Fw	TCGCGTTAAGCGGATGATGC	666	
*bla*_CTX-M25_	*bla*_CTX-M25_-Fw	GCACGATGACATTCGGG	327	
*bla*_CTX-M8/25_	*bla*_CTX-M1_-Rv	AACCCACGATGTGGGTAGC	666/327	

Fw: Forward, Rv: Reverse.

**Table 2:** Primers and conditions used for the amplification of the *mcr* genes.

Target	Primer	Sequence (5′–3′)	Amplicon Size (bp)	Conditions
*mcr-1*	*mcr-1*-Fw	CGGTCAGTCCGTTTGTTC	309	20 cycles of amplification at 94 °C for 30 s, 58 °C for 90 s, 72 °C for 1 min and final 72 °C for 10 min
	*mcr-1*-Rv	CTTGGTCGGTCTGTAGGG		
*mcr-2*	*mcr-2*-Fw	TGTTGCTTGTGCCGATTGGA	567	33 cycles of amplification at 95 °C for 3 min, 65 °C for 30 s, 72 °C for 1 min and final 72 °C for 10 min.
	*mcr-2*-Rv	AGATGGTATTGTTGGTTGCTG		
*mcr-3*	*mcr-3*-Fw	TTGGCACTGTATTTTGCATTT	542	30 cycles of amplification at 95 °C for 30 s, 50 °C for 30 s, 72 °C for 45 s and final 72 °C for 7 min.
	*mcr-3*-Rv	TTAACGAAATTGGCTGGAACA		
*mcr-4*	*mcr-4*-Fw	ATTGGGATAGTCGCCTTTTT	487	20 cycles of amplification at 94 °C for 30 s, 58 °C for 90 s, 72 °C for 1 min and final 72 °C for 10 min
	*mcr-4*-Rv	TTACAGCCAGAATCATTATCA		
*mcr-5*	*mcr-5*-Fw	ATGCGGTTGTCTGCATTTATC	1644	30 cycles of amplification at 95 °C for 30 s, 50 °C for 30 s, 72 °C for 95 s and final 72 °C for 5 min.
	*mcr-5*-Rv	TCATTGTGGTTGTCCTTTTCTG		

Fw: Forward, Rv: Reverse.

**Table 3:** Isolation of resistant bacteria in selective culture media (red: presence; green: absence). 1st SE, 2nd SE: first and second sampling events; ASE: Ebro Basin; ASC: Cantabrian Basin; ASA: Adour-Garonne Basin; ARDe: Discharge of wastewater entering the treatment plant; ARDs: Discharge of wastewater leaving the treatment plant; ARH: Discharge of hospital wastewater; ARM: Discharge of slaughterhouse wastewater; CPD (Cefpodoxime); FOX (Cefoxitin); VA (Vancomycin); CARB (Carbapenem); COL (Colistin).

Sample point	CPD 1st SE	CPD 2nd SE		FOX 1st SE	FOX 2nd SE		VA 1st SE	VA 2nd SE		CARB 1st SE	CARB 2nd SE		COL 1st SE	COL 2nd SE
1_ASE														
2_ASE														
3_ASE														
4_ASE														
5_ASE														
6_ASE														
7_ASE														
8_ASE														
9_ASE														
10_ASE														
11_ASE														
12_ASE														
13_ASE														
14_ASE														
15_ASE														
16_ASE														
17_ASE														
18_ASE														
19_ASE														
20_ASE														
21_ASC														
22_ASC														
23_ASA														
24_ASA														
25_ASA														
26_ASA														
27_ASA														
28_ASA														
29_ARD1e														
30_ARD1s														
31_ARD2e														
32_ARD2s														
33_ARD3e														
34_ARD3s														
35_ARD4e														
36_ARD4s														
37_ARH														
38_ARM														
39_ARM														
40_ARM														

**Table 4:** Percentage of positive rivers and WWTPs and collectors for each antibiotic in both sampling events and the number of resistant strains isolated.

Antimicrobial Resistance	% Positive Rivers		% Positive WWTP/C		N Total Isolates		N of Confirmed Target ARB (%)		Target ARB
	1st SE	2nd SE	1st SE	2nd SE	Rivers	WWTP/C	Rivers	WWTP/C	
Cefpodoxime	75	50	83.3	100	45	25	18 (40)	19 (75)	ESBL Enterobacteriaceae
Cefoxitin	71.4	75	83.3	100	89	39	2 (2.3)	0	*S. aureus* MRSA
Carbapenems	32.1	92.8 ^a^	41.6	91.6 ^a^	50	27	0	0	*A. baumanni*
							0	2 (7.4)	*P. aeruginosa*
							0	5 (18.5)	Enterobacteriaceae
Vancomycin	42.9	32.1	75	91.6	8	15	1 (12.5)	4 (26.6)	*E. faecium*
Colistin	3.6	53.6 ^b^	8.3	66.6 ^b^	8	5	2 (25)	2 (40)	Enterobacteriaceae
Total					200	111	23 (11.5)	32 (28.9)	

^a^ and ^b^ are the statistically significant differences (*p* < 0.05) between the 1st and 2nd sampling events (SE); WWTP/C: wastewater treatment plant and collectors; Target ARB: antibiotic resistant bacteria included in the list of high and critical priority pathogens of WHO.

**Table 5:** Characteristics and antibiotic resistance profile of isolated Gram negative bacteria.

Nº Strain	Samples Code	Species	Resistance Genes	AmpC	Antimicrobial Resistance										
					Penicillins	Cephalosporins	Monobactams	β-lactamase Inhibitors	Carbapenems	AminogLycosides	Quinolones	Tetracyclines	Others	MDR	Extensive MDR
1	3_ASE	*E. coli*	TEML-278, CTX-M14	-	AMP, PIP, MZ	CZ, CXM, CPD, CTX, CAZ, FOX, FEP	AZT	AMS	-	GM, TO	LV, CIP, MXF, NOR	TET	SXT, FOT, CHL	+	+
2	3_ASE	*E. coli*	TEML-278, CTX-M14	+	AMP, PIP, MZ	CZ, CXM, CPD, CTX, FOX, FEP	AZT	AMC, AMS	-	GM, TO	LV, CIP, MXF, NOR	TET	SXT, FOT, CHL	+	+
3	4_ASE	*E. coli*	TEML-278, CTX-M14	+	AMP, PIP, MZ	CZ, CXM, CPD, CTX, FOX, FEP	AZT	AMC, AMS	-	GM, TO	LV, CIP, MXF, NOR	TET	SXT, FOT, CHL	+	+
4	6_ASE	*E. coli*	CTX-M15	+	AMP, PIP, MZ	CZ, CXM, CPD, CTX, CAZ, FOX, FEP	AZT	AMC, AMS	MER	-	LV, CIP, MXF, NOR	TET, TIG	SXT, CHL	+	+
5	8_ASE	*E. coli*	TEML-278, CTX-M14	+	AMP, PIP, MZ	CZ, CXM, CPD, CTX, CAZ, FEP	AZT	AMS	-	-	LV, CIP, MXF, NOR	TET	-	+	+
6	17_ASE	*E. coli*	CTX-M1	-	AMP, PIP, MZ	CZ, CXM, CPD, CTX, CAZ, FEP	AZT	-	-	-	-	TET	CHL	+	+
7	17_ASE	*E. coli*	CTX-M1	-	AMP, PIP, MZ	CZ, CXM, CPD, CTX, CAZ, FEP	AZT	-	-	-	-	TET	CHL	+	+
8	29_ARD1e	*E. coli*	CTX-M1	+	AMP, PIP, MZ	CZ, CXM, CPD, CTX, CAZ, FEP	AZT	AMS	-	-	-	TET	-	+	+
9	30_ARD1s	*E. coli*	TEML-278, CTX-M14	-	AMP, PIP, MZ	CZ, CXM, CPD, CTX, CAZ, FEP	-	-	-	-	LV, CIP, MXF, NOR	-	-	+	-
10	30_ARD1s	*E. coli*	CTX-M14	+	AMP, PIP, MZ	CZ, CXM, CPD, CTX, CAZ, FOX, FEP	AZT	AMS	-	-	-	-	SXT, FOT	+	+
11	31_ARD2e	*E. coli*	CTX-M15	-	AMP, PIP, MZ	CZ, CXM, CPD, CTX, CAZ, FEP	AZT	-	-	-	-	-	SXT	+	-
12	32_ARD2s	*E. coli*	CTX-M1, SHV-12	-	AMP, PIP, MZ	CZ, CXM, CPD, CTX, CAZ, FEP	AZT	-	-	-	LV, CIP, MXF, NOR	-	CHL	+	+
13	32_ARD2s	*E. coli*	TEML-278, CTX-M1, SHV-12	-	AMP, PIP, MZ	CZ, CXM, CPD, CTX, CAZ, FEP	AZT	AMC, AMS, TZP	-	-	LV, CIP, MXF, NOR	TET	SXT, CHL	+	+
14	33_ARD3e	*E. coli*	TEM-171, CTX-M1	-	AMP, PIP, MZ	CZ, CXM, CPD, CTX, CAZ, FEP	AZT	AMS	-	-	-	-	-	+	-
15	34_ARD3s	*E. coli*	TEML-278, CTX-M14	-	AMP, PIP, MZ	CZ, CXM, CPD, CTX, CAZ, FEP	-	AMS	-	GM, TO	-	TET	SXT	+	+
16	34_ARD3s	*E. coli*	TEML-278, CTX-M14	-	AMP, PIP, MZ	CZ, CXM, CPD, CTX, CAZ, FEP	-	AMS	-	GM, TO	-	TET	SXT	+	+
17	35_ARD4e	*E. coli*	TEML-278, CTX-M1	-	AMP, PIP, MZ	CZ, CXM, CPD, CTX, CAZ, FEP	AZT	-	-	GM, TO	LV, CIP, MXF, NOR	TET	SXT, FOT	+	+
18	7_ASE	*E. coli*	TEML-278, SHV-12	-	AMP, PIP, MZ	CZ, CXM, CPD, CTX, CAZ, FEP	AZT	-	-	-	-	TET		+	-
19	30_ARD1s	*E. coli*	TEM-171, CTX-M15	-	AMP, PIP, MZ	CZ, CXM, CPD, CTX, CAZ, FEP	AZT	-	-	-	-	TET		+	-
20	32_ARD2s	*E. coli*	TEML-278, CTX-M15	-	AMP, PIP, MZ	CZ, CXM, CPD, CTX, CAZ, FEP	AZT	AMC, AMS	-	-	-	-	FOT	+	+
21	35_ARD4e	*E. coli*	TEM-176, CTX-M15, CTX-M14	-	AMP, PIP, MZ	CZ, CXM, CPD, CTX, CAZ, FEP	-	-	-	-	-	-		-	-
22	36_ARD4s	*E. coli*	OXA-1, CTX-M15	-	AMP, PIP, MZ	CZ, CXM, CPD, CTX, CAZ, FEP	AZT	AMC, AMS	-	TO	LV, CIP, MXF, NOR	-	SXT	+	+
23	40_ARM	*E. coli*	CTX-M15	-	AMP, PIP, MZ	CZ, CXM, CPD, CTX, CAZ, FEP	AZT	AMC, AMS	-	-	LV, CIP, MXF, NOR	TET	SXT, CHL	+	+
24	7_ASE	*S. fonticola*	CTX-M1	+	AMP, MZ	CZ, CXM, CPD, CTX, CAZ	AZT	AMC	-	-	-	-	-	+	-
25	11_ASE	*S. fonticola*	-	+	AMP	CZ, CXM, CPD, CTX	-	AMC	-	-	-	-	-	+	-
26	11_ASE	*S. fonticola*	CTX-M1	+	AMP	CZ, CXM, CPD, CTX, CAZ	AZT	AMC, AMS	-	-	-	-	-	+	-
27	11_ASE	*S. fonticola*	CTX-M15	+	AMP, PIP	CZ, CXM, CPD, CTX	-	AMC, AMS	-	-	MXF	-	FOT	+	+
28	20_ASE	*S. fonticola*	-	+	AMP, PIP, MZ	CZ, CXM, CPD, CTX, CAZ	AZT	AMC, AMS	-	-	-	-	-	+	-
29	3_ASE	*S. fonticola*	TEM-171	+	AMP	CZ, CXM, CPD, CTX	-	AMC	ETP	-	-	-	-	+	-
30	5_ASE	*S. fonticola*	TEM-171, ACC	+	AMP, PIP	CZ, CXM, CPD, CTX	-	AMC, AMS, TZP	-	-	-	-	-	+	-
31	11_ASE	*S. fonticola*	TEM-171	+	AMP	CZ, CXM, CPD, CTX	-	AMC	-	-	-	-	-	+	-
32	39_ARM	*S. fonticola*	CTX-M15, ACC	+	AMP, PIP, MZ	CZ, CXM, CPD, CTX	AZT	AMC, AMS	-	-	-	-	COL	+	+
33	8_ASE	*S. liquefaciens*	-	+	AMP	CZ, CXM, CPD	-	AMC	-	-	-	-	-	+	-
34	33_ARD3e	*K. pneumoniae*	TEM-171, SHV-12, CTX-M1, DHA	+	AMP, PIP, MZ	CZ, CXM, CPD, CTX, CAZ, FOX, FEP	AZT	AMC, AMS	MER	-	LV, CIP, MXF, NOR	TET	SXT, FOT, CHL	+	+
35	35_ARD4e	*K. pneumoniae*	CTX-M14	+	AMP, PIP, MZ	CZ, CXM, CPD, CTX, CAZ, FOX, FEP	AZT	AMC, AMS, TZP	MER	-	LV, CIP, MXF, NOR	TET	SXT, FOT, CHL	+	+
36	35_ARD4e	*K. pneumoniae*	TEML-278, SHV-12, OXA-1, CTX-M15	-	AMP, PIP, MZ	CZ, CXM, CPD, CTX, CAZ, FEP	AZT	AMC, AMS	-	TO	LV, CIP, MXF, NOR	-	SXT, CHL	+	+
37	11_ASE	*K. oxytoca*	-	+	AMP	CZ, FOX, FEP	AZT	AMC	-	-	-	-	-	+	-
38	33_ARD3e	*E. coli*	TEML-278, KPC	+	AMP, PIP, MZ	CZ, CXM, CPD, CTX, FOX, FEP	AZT	AMC, AMS, TZP	ETP, MER, IMP	-	LV, CIP, MXF, NOR	-	SXT, FOT	+	+
39	33_ARD3e	*K. oxytoca*	TEML-278, KPC	-	AMP, PIP, MZ	CZ, CXM, CPD, CTX, CAZ, FEP	AZT	AMC, AMS, TZP	ETP, MER, IMP	TO	LV, CIP, MXF, NOR	-	SXT, FOT	+	+
40	36_ARD4s	*C. freundii*	CTX-M1	+	AMP, PIP, MZ	CZ, CXM, CPD, CTX, CAZ, FOX, FEP	-	AMC, AMS, TZP	IMP	-	MXF	-	SXT	+	+
41	33_ARD3e	*C. freundii*	TEML-278, EBC, DHA, KPC	+	AMP, PIP, MZ	CZ, CXM, CPD, CTX, CAZ, FOX, FEP	AZT	AMC, AMS, TZP	ETP, MER, IMP	GM, TO	MXF	-	FOT	+	+
42	39_ARM	*C. freundii*	TEML-278, EBC, KPC	+	AMP, PIP, MZ	CZ, CXM, CPD, CTX, CAZ, FOX, FEP	AZT	AMC, AMS, TZP	ETP, MER, IMP	GM, TO	NOR	-	FOT	+	+
43	33_ARD3e	*P. aeruginosa*	TEML-278	-	AMP, PIP, MZ	CZ, CXM, CPD, CTX, CAZ, FOX, FEP	AZT	AMC, AMS	MER, IMP	AM, GM, TO	LV, CIP, NOR	TET	SXT, FOT, CHL	+	+
44	29_ARD1e	*P. aeruginosa*	-	+	AMP, MZ	CZ, CXM, CPD, CTX, FOX	-	AMC, AMS	ETP, MER, IMP	-	-	TET	SXT, FOT, CHL	+	+
45	22_ASC	*E. coli*	-	-	-	-	-	-	-	-	-	-	COL	-	-
46	39_ARM	*E. coli*	CTX-M15	-	AMP, PIP, MZ	CZ, CXM, CPD, CTX, CAZ, FOX, FEP	AZT	AMC, AMS, TZP	-	-	-	-	COL, FOT	+	+
47	40_ARM	*E. coli*	*mcr-1*	-	-	-	-	-	-	-	LV, CIP, MXF, NOR	TET	COL, SXT	+	-
48	1_ASE	*K. oxytoca*	-	-	AMP	-	-	-	-	-	-	-	COL, FOT	+	-

AMP, ampicillin; PIP, piperacillin; MZ, mezlocillin; CZ, cefazolin; CXM, cefuroxime; CPD, cefpodoxime; CTX, cefotaxime; CAZ, ceftazidime; FOX, cefoxitin; FEP, cefepime; AZT, aztreonam; AMC, amoxicillin-clavulanic acid; AMS, ampicillin-sulbactam; TZP, piperacillin-tazobactam; ETP, ertapenem; MER, meropenem; IMP, imipenem; GM, gentamicin; TO, tobramycin; LV, levofloxacin; CIP, ciprofloxacin; MXF, moxifloxacin; NOR, norfloxacin; TET, tetracycline; TIG, tigecycline; SXT, trimethoprim-sulfamethoxazole; COL, colistin; FOT, fosfomycin; CHL, chloramphenicol.

**Table 6:** Characteristics and antibiotic resistance profile of isolated Gram positive bacteria.

Nº Strain	Samples Code	Species	Resistance Genes	Antimicrobial Resistance										
				Penicillins	Cephalosporins	Carbapenems	Glycopeptides	Aminoglycosides	Macrolides	Quinolones	Tetracyclines	Others	MDR	Extensive MDR
49	14_ASE	*E. faecium*	*vanB*	OX	CXM, FEP	ETP, MER	VA	GM	ERY	-	TET	FA, Cd, FOT, SYN	+	+
50	40_ARM	*E. faecium*	*vanB*	-	CXM, FEP	-	VA	-	ERY	-	-	FA, MUP, DAP, Cd, FOT	+	+
51	29_ARD1e	*E. faecium*	*vanB*	OX	CXM, FEP	ETP, MER	VA	GM, TO	ERY	LV, CIP, MXF	TET	FA, SXT, Cd, FOT, SYN	+	+
52	33_ARD3e	*E. faecium*	*vanB*	P, OX	CXM, FEP	ETP, MER	VA	GM, TO	ERY	LV, CIP, MXF	TET	FA, SXT, DAP, Cd, FOT, RA, FD, SYN	+	+
53	40_ARM	*E. faecium*	*vanB*	OX	CXM, FEP	ETP, MER	VA	GM	ERY	LV, CIP, MXF	TET	FA, SXT, Cd, FD, SYN	+	+
54	4_ASE	*S. aureus*	*-*	AMP, P, OX	CXM, FOX, FEP	ETP, MER, IMP	-	-	AZI, ERY	-	-	MUP, Cd, FOT	+	+
55	15_ASE	*S. aureus*	-	AMP, P, OX	FOX	-	VA, TEC	-	ERY	-	-	LZ, DAP, Cd, SYN	+	+

AMP, ampicillin; P, Penicillin; OX, oxacilin; CXM, cefuroxime; FOX, cefoxitin; FEP, cefepime; ETP, ertapenem; MER, meropenem; IMP, imipenem; VA, vancomycin; TEC, teicoplanin GM, gentamicin; TO, tobramycin; AZI, azithromycin; ERY, erythromycin; LV, levofloxacin; CIP, ciprofloxacin; MXF, moxifloxacin; TET, tetracycline; FA, fusidic acid; SXT trimethoprim-sulfamethoxazole; Lz, Linezolid; MUP mupirocin; DAP, Daptomycin; Cd, clindamycin; FOT, fosfomycin; RA, rifampicin; FD, nitrofurantoin; SYN, synercid.

## Supplementary Materials

The following are available online at https://www.mdpi.com/2076-2607/8/9/1425/s1, Table S1. Characteristics of sampling points in rivers for the north of Spain and south of France. Table S2. Characteristics of sampling points for sewage water in the Navarra Region. Table S3. Zone diameter breakpoints for the different antibiotics tested.
