# Supplementary material for: Multidrug-Resistant Bacteria Isolated from Different Aquatic Environments in the North of Spain and South of France
Source: Microorganisms. 2020 Sep 16;8(9):1425. doi: 10.3390/microorganisms8091425 (PMC7565385; doi:10.3390/microorganisms8091425)
Supplement: Supplementary file 1 [file microorganisms-08-01425-s001.pdf]

Table S1. Characteristics of sampling points in rivers (north of Spain and south of France). Data supplied by the Laboratory of Water and Environmental Health from University of Zaragoza.

Code	River	Location (country)	Cattle Exploitations						
			Porcine	Poultry	Rabbits	Ovine	Bovine	Equine	Caprine
1_ASE	Segre	Lleida (Spain)	x	x	-	-	-	-	-
2_ASE	Noguera Ribagorzana	Lleida (Spain)	x	x	x	-	-	-	-
3_ASE	Clamor Amarga	Huesca (Spain)	x	x	x	-	x	-	-
4_ASE	Cinca	Huesca (Spain)	x	x	x	-	-	-	-
5_ASE	Alcanadre	Huesca (Spain)	x	x	-	-	-	-	-
6_ASE	Flumen	Huesca (Spain)	x	x	x	-	x	-	-
7_ASE	Gállego	Zaragoza (Spain)	x	x	-		x	-	-
8_ASE	Arba de Riquel	Zaragoza (Spain)	x	-	-	x	-	-	-
9_ASE	Aragon subordan	Huesca (Spain)	-	-	-	x	-	x	x
10_ASE	Aragón	Navarra (Spain)	x	x	-	-	-	x	-
11_ASE	Irantzu	Navarra (Spain)	x	x	-	-	-	x	-
12_ASE	Arakil	Navarra (Spain)	x	x	-	x	-	x	x
13_ASE	Queies	Zaragoza (Spain)	x	x	-	-	-	x	-
14_ASE	Alhama	La Rioja (Spain)	x	x	-	-	-	x	-
15_ASE	Ega	Navarra (Spain)	x	x	-	-	-	x	-
16_ASE	Ega	Navarra (Spain)	x	x	-	-	-	x	-
17_ASE	Arga	Navarra (Spain)	x	x	-	x	x	x	-
18_ASE	Arga	Navarra (Spain)	x	x	-	x	x	x	-
19_ASE	Ebro	Navarra (Spain)	x	x	x	-	-	x	-
20_ASE	Ebro	Navarra (Spain)	x	x	x	-	-	x	-
21_ASC	Bidasoa	Navarra (Spain)	x	-	-	x	-	-	x
22_ASC	Bidasoa	Navarra (Spain)	x	-	-	x	-	-	x
23_ASA	L´Adour	Pyrénées-atlantiques (France)	x	x	-	x	-	-	x
24_ASA	Le Gabas	Pyrénées-atlantiques (France)	x	x	-	-	-	-	-
25_ASA	La Garonne	Haute-Garonne (France)	-	x	x	-	-	-	-
26_ASA	La Save	Haute-Garonne (France)	x	x	x	-	-	-	-
27_ASA	L´Echez	Haute-Pyrénées (France)	x	x	x	-	-	-	-
28_ASA	La Baïse	Haute-Pyrénées (France)	x	x	x	-	-	-	-

ASE: Surface water of the Ebro basin. ASC: Surface water of the Cantabrian basin. ASA / G: Surface water of the Adour-Garonne basin.

Table S2. Characteristics of sampling points for sewage water in the Navarra Region. Data supplied by the Laboratory of Water and Environmental Health from University of Zaragoza.

**Code**	**Sampling point**	**Location**	**Inhabitants**	**River receiver**	**Type of Waste**
29_ARD1e	WWTP (influent)	Estella	90.000	Ega	Urban
30_ARD1s	WWTP (effluent)				Urban
31_ARD2e	WWTP (influent)	Pamplona	550.000	Arga	Urban
32_ARD2s	WWTP (effluent)				Urban
33_ARD3e	WWTP (influent)	Tudela	82.500	Ebro	Urban
34_ARD3s	WWTP (effluent)				Urban
35_ARD4e	WWTP (influent)	Bera	4.900	Bidasoa	Urban
36_ARD4s	WWTP (effluent)				Urban
37_ARH	Hospital collector	Tudela	-	Ebro	Hospital
38_ARM	Slaughterhouse collector		-		Slaughterhouse (birds)
39_ARM	Slaughterhouse collector	Bera	-	Bidasoa	Slaughterhouse (ducks)
40_ARM	Slaughterhouse collector	Murchante	-	Ebro	Slaughterhouse (rabbits)

ARDe: Discharge of wastewater entering the treatment plant. ARDs: Discharge of wastewater leaving the treatment plant. ARH: Discharge of hospital wastewater.ARM: Discharge of slaughterhouse wastewater.

Table S3. Zone diameter breakpoints for the different antibiotics tested.

**Antibiotic**	**Zone diameter breakpoints (mm)**			**Microorganism**	**Guideline**
	S	I	R		
Cefotaxime	≥ 26	23-25	≤22	*Enterobacteriaceae*	CLSI
Ceftazidime	≥ 21	18-20	≤17		
Ceftriaxone	≥ 23	20-22	≤19		
Aztreonam	≥ 21	18-20	≤17		
Amox-clavulanate	≥ 18	14-17	≤13		
Ertapenem	≥ 22	19-21	≤18		
Imipenem	≥ 23	20-22	≤19		
Meropenem	≥ 23	20-22	≤19		
Ertapenem	ND	ND	ND	*P. aeruginosa*	CLSI
Imipenem	≥ 19	19-18	≤15		
Meropenem	≥ 19	19-18	≤15		
Ertapenem	ND	ND	ND	*A. baumanii*	CLSI
Imipenem	≥ 22	19-21	≤18		
Meropenem	≥ 18	15-17	≤14		
Cefoxitin	≥ 22	-	≤21	*S. aureus*	CLSI
Antibiotic	MIC Breakpoints (μg/mL)			Microorganism	Guideline
	S	I	R		
Colistin	≤2	-	≥ 2	*Enterobacteriaceae*	EUCAST
Vancomycin	≤4	8-16	≥ 32	*E. faecium*	CLSI
Teicoplanin	≤8	16	≥ 32		
